# Investigating the Sudan virus outbreak in Uganda through the deployment of a mobile laboratory

**DOI:** 10.1186/s12879-026-12556-8

**Published:** 2026-01-16

**Authors:** Godfrey Pimundu, Tonny Muyigi, Eunice Jennifer Nambozo, Christopher Okiira, Benedict Kanamwanji, Rebecca Nalwanga, Joseph Sekate, Raymond Mugabe, Juliet Naiga, Isaac Sewanyana, Julius Lutwama, Stephen Balinandi, Pontiano Kaleebu, Jane Ruth Aceng, Diana Atwiine, Henry Mwebesa, Henry Kyobe Bosa, Atek Kagirita, Charles Olaro, Andrew Nsawotebba, Emmanuel Achol, Hakim Lagu, Eric Nzeyimana, Juergen May, Florian Gehre, Muna Affara, Susan Nabadda

**Affiliations:** 1 National Health Laboratory and Diagnostics Services, Ministry of Health, Kampala, Uganda; 2 Mubende Regional Referral Hospital, Mubende, Uganda; 3 East African Community Secretariat, Arusha, Tanzania; 4 Bernhard-Nocht-Institute for Tropical Medicine, Hamburg, Germany; 5 Uganda Virus Research Institute, Entebbe, Uganda; 6 Ministry of Health, Kampala, Uganda; 7 Infectious Diseases Research Collaboration (IDRC), Kampala, Uganda

**Keywords:** Sudan virus (SUDV), Ebola virus (EBOV), Mobile laboratory, Disease outbreak

## Abstract

**Background:**

Uganda faced its sixth reported Ebola outbreak between September 2022 and January 2023, the fifth to be recorded as Sudan Virus Disease (SVD) in the country. In response to this, the Ugandan Ministry of Health (MoH), rapidly deployed a mobile laboratory from the National Health Laboratory and Diagnostics Services (NHLDS) within one week of the declared outbreak. Here we describe the deployment of the mobile laboratory to Mubende, the outbreak epicentre, as part of the national response. This provided (1) efficient diagnostics and characterization of Sudan virus cases to support national data reporting (2), greater insight into Sudan virus kinetics, including viral clearance and risk factor analysis and (3) evaluation of the integration of the mobile laboratory into the national outbreak response.

**Methods:**

The mobile laboratory was deployed to the Mubende Regional Referral Hospital and positioned next to the established Ebola Treatment Unit (ETU). The laboratory was deployed for 177 days, in continuous operation by a team of 18 trained personnel. Molecular testing, using reverse-transcriptase polymerase chain reaction (RT-PCR) was carried out on all samples received in the laboratory for both Sudan virus diagnosis and differential diagnosis for other viral haemorrhagic fever (VHFs). All results from the mobile laboratory were fed directly into the national database, to enable a coordinated response to the outbreak and analysis of the outbreak data on a national level.

**Results:**

Nationwide, there were 142 confirmed cases, 55 deaths and 22 probable cases reported in the outbreak. During the mobile laboratory deployment, 3282 samples were tested and 72 SVD cases confirmed by RT-PCR, with an average turn-around-time (TAT) of 6 h. In addition to molecular diagnostic confirmation of suspect cases, the mobile laboratory functioned to support follow-up surveillance of Sudan virus survivors (4 breast feeding mothers and 22 males). Sudan virus RNA was found in the breast milk a median of 135 days after initial test positivity and in the semen of male survivors median 176 days later. We observed the highest risk for contracting the disease in health care workers and a significant correlation between patient viral load at initial diagnosis and patient outcome. Differential diagnosis of other VHFs in the mobile laboratory and at the Uganda Virus Research Institute (UVRI) identified 6 Rift Valley fever (RVF) and 7 Crimean-Congo haemorrhagic fever (CCHF) cases co-circulating in the current SVD outbreak.

**Conclusion:**

Having the mobile laboratory stationed next to the ETU at the epicentre of the outbreak, markedly reduced diagnostic turn-around-time (TAT) and improved interoperability between the laboratory and the ETU, supporting containment and treatment. Furthermore, integration of the mobile laboratory into existing national outbreak systems, ensured rapid data provision for daily decision making by the national task force. The data from this study contributes to a greater understanding of Sudan virus.

**Clinical trial number:**

Not applicable.

## Background

Ebola disease, resulting from viruses within the *Filoviridae* family, cause severe, potentially fatal systemic disease in humans and non-human primates [1]. To date, four species of Ebola virus have been identified to cause disease in humans: Ebola virus (EBOV), Sudan virus (SUDV), Tai Forest virus (TAFV) and Bundibugyo virus (BDBV) [2]. Transmission is through direct contact with the blood, bodily secretions, and other biological materials of infected sick or dead humans, non-human primates, bats or other infected mammals. Ebola patients present most frequently with fever, asthenia, diarrhoea, abdominal pain, headache, arthralgia, myalgia, sore throat, dysphagia, and conjunctivitis, making clinical diagnosis often challenging due to the unspecific nature of the symptoms. Therefore, rapid confirmation by laboratory diagnostic methods is critical to initiate supportive treatment and containment measures.

Since first identified in the Democratic Republic of the Congo (DRC) in 1976, the majority of Ebola cases have occurred in central Africa, in the countries of Gabon, the DRC, and the Republic of the Congo [2, 3]. The West African outbreak of 2013–2016 was the largest Ebola outbreak in history. It originated in Guinea in December 2013 and later spread to neighbouring West African countries, including Sierra Leone and Liberia, resulting in 28,652 human infections and 11,325 [4]. The location and scale of the 2013–2016 outbreak was entirely unexpected, underscoring the importance of early detection and control measures in communities.

Uganda has previously had five outbreaks of Ebola disease from two species: Sudan virus (4 outbreaks) and Bundibugyo virus [5, 6]. Importation of Ebola virus (formally Zaire ebolavirus) from the neighbouring DRC was reported in 2018, however, there was no in-country transmission [5]. The first ever reported Ebola disease outbreak in Uganda was on October 8, 2000, in the Gulu district of the country and was confirmed as Sudan virus disease (SVD). This was the largest Ebola outbreak in the country (425 cases and 224 deaths), with a case fatality rate (CFR) of 53% (similar to previously reported rates for SUDV) [7, 8]. In subsequent years since 2000, four smaller Ebola disease outbreaks have followed in Uganda, in the district of Bundibugyo in 2007, Luweero in 2011 and 2012 and Kibaale in 2012 [5]. More recently, Uganda has experienced its seventh Ebola disease outbreak in January 2025, which was also confirmed as SVD [9]. With the outbreaks often occurring in remote locations and testing of risk group 4 pathogens restricted to a limited number of centralized facilities in the country, such outbreaks can be logistically difficult to manage. Both sample integrity and turnaround time (TAT) for laboratory confirmed disease diagnosis are impacted by long delays in sample shipment to centralized facilities, thereby contributing to the challenges associated with EVD outbreak response and containment efforts.

On the 20th September 2022, the Ministry of Health (MoH) of Uganda declared the sixth Ebola outbreak originating in the country, located in the Mubende district and confirmed as caused by Sudan virus [10]. In an effort to reduce laboratory diagnostic TAT and support more rapid containment efforts, the MoH decided to deploy a modular mobile laboratory as part of the national Ebola outbreak response measures. The mobile laboratory was deployed to the epicentre of the outbreak on the 28th September 2022 (one week after the outbreak was declared), and positioned at the Mubende Regional Referral Hospital, directly next to the Ebola Treatment Unit (ETU). At this stage in the outbreak, 26 cases had been confirmed by the Uganda Virus Research Institute (UVRI), and there was an urgent need for surge capacity to support containment efforts. Here, we describe the role of the mobile laboratory in supporting the national response to the SVD outbreak in Uganda between September 2022 and January 2023, as well as the integration of the mobile laboratory with existing laboratories mandated with VHF outbreak diagnostics in Uganda. The deployment of the mobile laboratory provides an opportunity to evaluate the effectiveness of interoperability between Mobile Laboratories and ETUs in an outbreak response. In addition, given the large patient numbers tested in the mobile laboratory, at the Uganda Virus Research Institute (UVRI) and the Central Public Health Laboratory (CPHL) during the outbreak period, the epidemic provided a rare opportunity to gain greater understanding of the clinical virology of SUDV.

## Methods

### Study location

The mobile laboratory was deployed from the National Health Laboratory Diagnostic Services (NHLDS) to the Regional Referral Hospital (RRH) in Mubende town, situated in the Mubende district. Mubende town is approximately 150 km by road west of Kampala, the capital of Uganda, and was the epicentre of this SVD outbreak; with the mobile laboratory positioned directly next to the established ETU. Samples were referred to the mobile laboratory for testing, from both the ETU within the hospital and all districts within approximately 100 km from Mubende, including Kassanda, Mityana, Bunyangabo, Kakumiro and Kagadi. Suspect samples from Kampala were sent to the Central Public Health Laboratory (CPHL) for testing and samples from other districts within Uganda went to UVRI in Entebbe.

### Study population

The study included confirmed, probable, or suspected cases of SVD in both alive and deceased patients of any age or sex. The standard case definition for an SVD suspect, probable, and confirmed case served as the basis for the identification of the patients [11].

### Staffing schedule in the mobile laboratory

The mobile laboratory was operated by a team of 18 trained personnel throughout the 177 days of the outbreak deployment. Due to the urgency of laboratory results to inform both patient care and the district and national task forces on the next steps in outbreak response activities, the laboratory personnel worked in shifts over 24-hour periods, to ensure continuous diagnosis of cases in the fastest possible TAT. The mobile laboratory processed an average of 25 samples per day. One limitation was sample throughput is the mobile glovebox, which can comfortably process a maximum of 12 samples per batch. Therefore, the mobile laboratory was equipped with two mobile gloveboxes to ensure that all samples received into the mobile laboratory were processed within the same day. The mobile laboratory was operated in line with standardized mobile laboratory procedures for the containment and diagnosis of risk group 4 pathogens. Access to the laboratory was restricted exclusively to the trained laboratory personnel and compliance strictly followed, to assure biosafety and biosecurity throughout the entire mobile laboratory deployment.

### Sample collection and processing

Clinical samples were collected from suspected, probable, and confirmed cases that met the standard Ebola disease case definition guideline criteria [11]. A dedicated team of sample collectors obtained the samples from the field, hospitals and ETUs (including blood, body fluids, breast milk, semen and buccal cavity swabs from the deceased) and delivered them to the mobile laboratory. Breast milk and semen samples collect for the initial screening test, were only collected from those suspects meeting specific criteria, such as lactating mothers. All samples received in the mobile laboratory for analysis were tested, regardless of sample type. Laboratory staff were dressed in appropriate PPE to safely receive the samples. Surface disinfection of all received samples was carried out with a 0.5% hypochlorite solution prior to crosschecking; they were correctly triple-packed. Any sample information queries were immediately cross checked with the sample collection team, before processing in the laboratory. In the rare case of miss-labelling (there was one incident in the mobile laboratory), the laboratory personnel followed the ISO15189 and SOP procedures for managing incidence and nonconformities.

All PCR positive samples were archived in duplicates and triple-packed in bio bottles using 2 ml screw-capped tubes and stored temporarily in the laboratory at -20 °C for less than 2 weeks. For long-term storage samples were shipped to UVRI through the central national repository at the National Health Laboratory and Diagnostic Services (NHLDS). 20% of the negative samples were archived in the same manner as the positives, except that they were packed in cryo-boxes, as defined by the national Uganda quality management system. The remaining samples were destroyed with 0.5% bleach and discarded. All of the procedures were carried out in a mobile containment unit (glovebox), under negative pressure. Confirmatory diagnosis of the first 20 positive and negative cases took place at UVRI, ensuring diagnostic concordance and that a robust national mechanism was in place to assure data quality.

### Sample reception into the mobile laboratory, sample inactivation and nucleic acid extraction

Triple-packed samples were opened under 0.5% hypochlorite solution to gain access to the secondary container, allowing for a 10-minute contact time. The secondary container was then imported for inactivation into the BSL-3 containment unit’s glovebox. Qiagen reagents containing viral lysis buffer (Qiagen, Hamburg, Germany), followed by addition of molecular grade ethanol were used for chemical inactivation. The inactivated samples were removed from the glovebox for subsequent manual extraction with the Qiagen Virus RNA Mini kit. Extracted RNA from samples was either used immediately or stored at -20 °C for Real Time Polymerase Chain Reaction (RT-PCR) amplification.

### Reverse transcription polymerase chain reaction assays

#### Sudan virus detection

Filovirus amplification was performed in a real time thermal cycler (Bio-rad CFX 96 Dx thermocycler system) using three detection or typing kits namely; Altona RealStar Filovirus Screen RT-PCR Kit 1.0 (Altona Diagnostics GmbH, Hamburg, Germany), Altona RealStar Filovirus Type RT-PCR Kit 2.0, (Altona Diagnostics Hamburg, Germany), and bioperfectus Ebola virus real time PCR kit (Jiangsu Bioperfectus Technologies Co, Ltd, China). For each of the kits, a 20 µl master mix was prepared in a nuclease free environment to which 10ul or 5ul of the extracted RNA/template was added for real time amplification. Clinical field evaluation of the Altona RealStar Filovirus Screen RT-PCR kit 2.0 and the Bioperfectus Ebola virus real time PCR kit was carried out in the mobile laboratory. The clinical field performance and LoD determination (as expressed in relative RNA concentrations or Log10 RNA dilutions per µl of RT-PCR reaction) of the two assays demonstrated that both assays were robust in detecting SUDV positive samples in a wide range of relative RNA concentrations with good linearity between relative RNA concentrations and Ct values (bioPerfectus LoD50 of 10-6.68 *versus* altona filovirus screen LoD50 of 10-6.07) (manuscript in preparation).

The PCR amplification conditions were set up according to manufacturer’s instructions as follows for the different types on amplification kits; Altona Filovirus Screen and Filovirus Type (reverse transcription – 20 min at 55 °C, denaturation – 2 min at 95 °C and amplification – 15 s at 95 °C, 45 s at 58 °C, 15 s at 72 °C); bioperfectus (reverse transcription – 10 min at 50 °C, initial denaturation – 5 min at 95 °C, denaturation – 10 s at 95 °C and annealing, extension, fluorescent signal collection – 30 s at 60 °C). RT-PCR results were interpreted using the Bio-Rad CFX Manager Dx software. Both heterogenous and endogenous controls were used for quality controlling the assay. A negative result or viral clearance in a patient sample was classified as when the cycle threshold (Ct) value was no longer detectable. Ct values were used as a semiquantitative proxy for RNA levels, with lower Ct values corresponding to higher viral load.

#### VHF differential diagnosis

A subset of individuals presenting with viral haemorrhagic fever (VHF) symptoms, tested negative for SUDV and were subsequently tested by differential diagnosis for Rift Valley fever (RVF) and Crimean-Congo haemorrhagic fever (CCHF) and yellow fever (YF), following the VHF PCR testing algorithm of Uganda. The mobile laboratory used the RealStar^®^ Rift Valley Fever Virus RT-PCR Kit 1.0, RealStar^®^ CCHFV RT-PCR Kit 1.0, and The RealStar^®^ Yellow Fever Virus RT-PCR Kit 1.0 in accordance with manufacturer’s instructions, whereas UVRI used CDC custom primers and probes [12].

### Breastmilk and semen samples testing, the survivors’ program

It is a requirement in the response process that the survivors’ management program is initiated. To evaluate the persistence of SUDV after infection in female and male survivors, breast milk and semen samples were taken at regular time points and analyzed by PCR for the presence of Sudan virus RNA. Breast milk and semen samples were collected at regular intervals from all survivors between January 11, 2023 – beginning of March 2023. In total four [4] breast feeding mothers and twenty-two [22] sexually active male survivors were monitored until they either repeatedly tested negative by RT-PCR or obtaining the samples was no longer possible. All samples were processed in the mobile laboratory using the SUDV diagnostic PCR procedures. The survivor analysis of the four mothers and 22 males was continued at UVRI when the mobile laboratory deployment ended in March 2023.

### Statistical analysis

Data was collected in Microsoft Excel (Microsoft Corporation, Redmond, WA, USA). Statistical analysis was carried out using StataSE and Graphpad PRISM version 9, including calculations of frequencies and proportions. Categorical data are presented as numbers (percentages), while continuous data are reported as medians, with interquartile range (IQR) as appropriate. Analysis was carried out to determine demographic characteristics of the study population, including age, gender and occupation. Non-parametric Mann-Whitney tests were used due to the relatively small sample size, to assess statistical significance between clinical outcome and viral load, with Ct values used as a proxy for viral load. A p-value of less than 0.05 was considered statistically significant. Survival analysis of viral RNA persistence in breast milk and semen, were carried out using Graphpad Prism version 9.

### Ethics

This work was done to respond to an outbreak of SVD, or mandated surveillance, and no ethical review or personal consent from sample donors was required. The Ministry of Health of Uganda considers these activities as events of significant public health importance that require immediate response and containment.

## Results

During the declared outbreak period between 20th September 2022 and 11th January2023, a total of 6723 samples were received for SUDV testing at the designated Ministry of Health Laboratories in Uganda; (1) the Central Public Health Laboratory (CPHL) located in Kampala (2), the East African Community (EAC) mobile laboratory positioned in the outbreak epicentre at Mubende Regional Referral Hospital and (3) the Uganda Virus Research Institute (UVRI). The majority of samples received in the laboratories were whole blood samples (5254), however, swabs from suspect cases and deceased individuals, as well as semen, urine, breast milk and other body fluids were also processed dependent on meeting certain criteria (see Table 1). Of the samples tested nationally, a total of 142 individuals were confirmed SUDV-positive during the outbreak period, of which 72 SUDV-positive cases were diagnosed within the mobile laboratory in Mubende. No breast milk or serum samples tested positive during initial screening, although positivity in these sample types was observed during the survivor followup analysis. The distribution of sample types received in the Mubende mobile laboratory reflected those received nationally, with over 48.8% of all SUDV positive cases identified in the mobile laboratory (Table 1).

**Table 1:** Samples received in the laboratory for initial SUDV testing during the outbreak in Uganda

Sample type	Total sample tested nationally (Mubende, CPHL, UVRI)	Sample no. Mubende mobile lab	PCR positive tests nationally (Mubende, CPHL, UVRI)	PCR positive tests Mubende mobile lab
			SUDV	SUDV
ANAL SWAB	3	3	0	0
ASPIRATE	3	3	0	0
BODY FLUID	94	94	0	0
BREAST MILK	23	23	0	0
BUCCAL SWAB	24	24	0	0
CSF	1	0	0	0
SWABS (buccal cavity of deceased)	122	6	0	0
SWAB (Oral / Nasal)	71	30	3	1
SWAB (other)	1053	375	0	0
SEMEN	33	21	0	0
SERUM	15	0	0	0
UNKNOWN	27	27	3	3
WHOLE BLOOD	5254	2,676	136	68
TOTAL	6723	3282	142	72

### SUDV outbreak characteristics

We investigated the demographic characteristics of the SVD cases both nationally and specifically the positive cases analysed in the Mubende mobile laboratory. The gender distribution between the Mubende mobile laboratory and those observed nationally were proportionally the same, see Table 2. We also observed a similar age distribution of positive cases between the diagnostic labs, with the most represented age group being 19–34 years (45.1%). When analysing occupation, we observed an under representation of positive cases from individuals within the health sector presenting at the Mubende mobile laboratory (6.6% vs. 17.9%). For other sectors, the distribution was similar, with farming being the most common occupation. All patients testing positive in the Mubende laboratory fell ill in the districts of Kasanda and Mubende. Patients falling ill in other districts were served by the other laboratories mandated in the outbreak diagnostics.

**Table 2:** Characteristics of the positive SVD cases in the outbreak

Characteristic	Mubende mobile laboratory *n* = 72	Other national labs *n* = 70	National total *n* = 142
**Gender**			
Male	44 (61%, 95% CI: 49–72)	40 (57%, 95% CI: 45–68)	84 (59%, 95% CI: 51–67)
Female	28 (39%, 95% CI: 28–51)	30 (43%, 95% CI: 32–55)	58 (41%, 95% CI: 33–49)
**Age (years)**			
0–5	4 (5.6%, 95% CI: 2–14)	3 (4.3%, 95% CI: 1–12)	7 (4.9%, 95% CI: 2–10)
6–18	9 (12.5%, 95% CI: 7–22)	11 (15.7%, 95% CI: 9–26)	20 (14.1%, 95% CI: 9–21)
19–34	35 (48.6%, 95% CI: 37–61)	29 (41.4%, 95% CI: 30–53)	64 (45.1%, 95% CI: 37–53)
35–64	22 (30.5%, 95% CI: 21–43)	27 (38.6%, 95% CI: 28–51)	49 (34.5%, 95% CI: 27–43)
65+	1 (1.4%, 95% CI: 0–8)	0 (0.0%)	1 (0.7%, 95% CI: 0–4)
Unknown	1 (1.4%, 95% CI: 0–8)	0 (0.0%)	1 (0.7%, 95% CI: 0–4)
**Occupation**			
Health Care	4 (6%)	12 (17%)	16 (11%)
Business	12 (17%)	8 (11%)	20 (14%)
Child / Student	7 (10%)	14 (20%)	21 (15%)
Miner	1 (1%)	0 (0%)	1 (1%)
Farmer	28 (39%)	29 (41%)	57 (40%)
Unknown / Other	20 (28%)	7 (10%)	27 (19%)
**District where patient fell ill**			
Bunyangabu	–	1 (1%)	1 (1%)
Jinja	–	2 (3%)	2 (1%)
Kampala	–	12 (17%)	12 (8%)
Kasanda	39 (54%)	5 (7%)	44 (31%)
Kyegegwa	–	3 (4%)	3 (2%)
Mpigi	–	1 (1%)	1 (1%)
Mubende	25 (35%)	36 (51%)	61 (43%)
Wakiso	–	6 (9%)	6 (4%)
Other districts	8 (11%)	4 (6%)	12 (8%)

Focusing on the Mubende mobile laboratory, where we had available outcome data for the patients, we observed higher fatality among females (53.6%) than males (38.6%), though not statistically significant (*p* ≈ 0.2, Fisher’s exact test) (see Table 3). When analysing case fatality relative to age group, we observed mortality increased with age, peaking among adults aged 35–64 years (68.2%, 95% CI: 50.0–86.4).

**Table 3:** Case fatality rates (CFR) among SVD patients by sex and age group in the Mubende mobile laboratory (*n* = 72)

Category	Group	*n*	Deaths (*n*)	CFR (%) [95% CI]
Sex	Male	44	17	38.6% (25.0–52.3)
	Female	28	15	53.6% (35.7–71.4)
Age (years)	0–5	4	1	25.0% (0.0–75.0)
	6–18	9	4	44.4% (11.1–77.8)
	19–34	35	11	31.4% (17.1–48.6)
	35–64	22	15	68.2% (50.0–86.4)
	≥ 65	1	0	0.0% (0.0–0.0)
	Unknown	1	0	0.0% (0.0–0.0)

### Geographical location of the SUDV epidemic and concurrent viral haemorrhagic outbreaks nationally

**Fig. 1:**
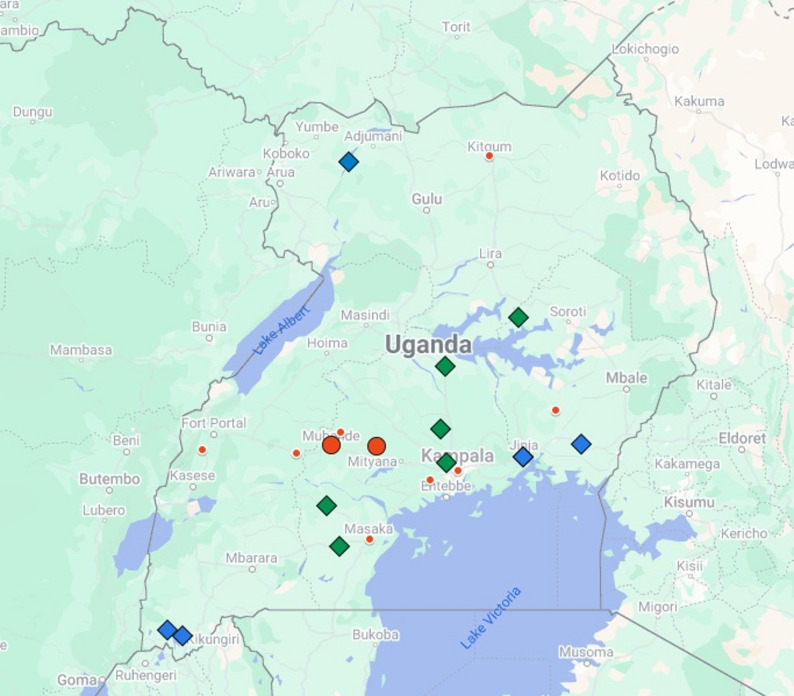
Map of positive SUDV, RVF and CCHF cases. Positive SUDV cases are denoted by red circles, positive RVF cases by blue diamonds and positive CCHF by green diamonds. The larger red circles represent the epicentre of the SDV outbreak, where the majority of cases were confirmed (Mubende 65 cases and Kasanda 48 cases). There was also a concentration of confirmed cases in the capital, Kampala (18 cases)

In total 1633 differential diagnosis tests were conducted (12 in the mobile lab and the rest at UVRI). This revealed 6 RVF positive cases and 7 CCHF positive cases, demonstrating simultaneous VHF outbreaks occurring within the same time period. Geospatial mapping of the districts where patients fell ill, revealed that the majority of SUDV cases were from two districts [1], Mubende district (the epicentre with 65 confirmed cases) and Kasanda district (48 confirmed cases) (see Fig. 1). A further 18 cases were clustered in the Kampala district, with the remaining SVD cases located close to the Mubende epicentre and to Kampala. In contrast, the RVF cases were geographically dispersed, with three cases located in the South Western districts of Kabale and Rubanda, one in Northern Uganda in the Obongi district and two in the Eastern districts of Jinja and Bugiri. The CCHF cases were located within the South Western to North Eastern cattle corridor [13], in the districts of Wakiso, Kaberamaido, Ssembabule, Lwengo, Nakaseke and Nakasongola.

### Diagnostic testing in the Mubende mobile laboratory

Of the 6723 samples received during the outbreak, 3282 were processed by the mobile laboratory in Mubende (Table 1). Samples were received from both the ETU, located next to the laboratory in Mubende district hospital and from suspect cases within the surrounding community, with a daily load of approximately 25 samples. TAT from sample reception to result submission to the Ministry of Health (MoH) Results Dispatch system (RDS), was on average 6 h (markedly reduced from the centralized testing system, which can take in excess of 24 h.

**Fig. 2:**
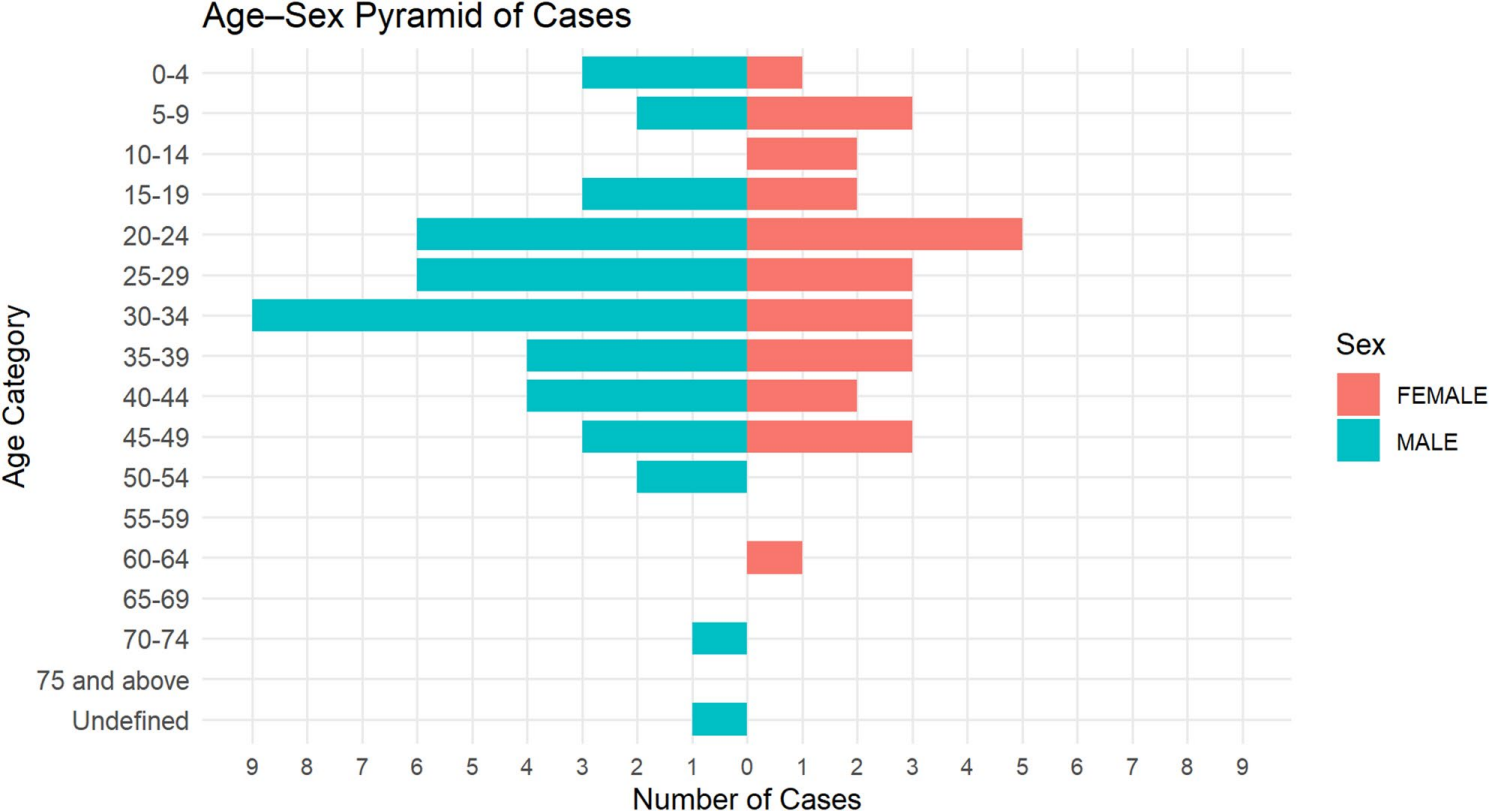
Age-Sex distribution of SVD positive cases

**Fig. 3:**
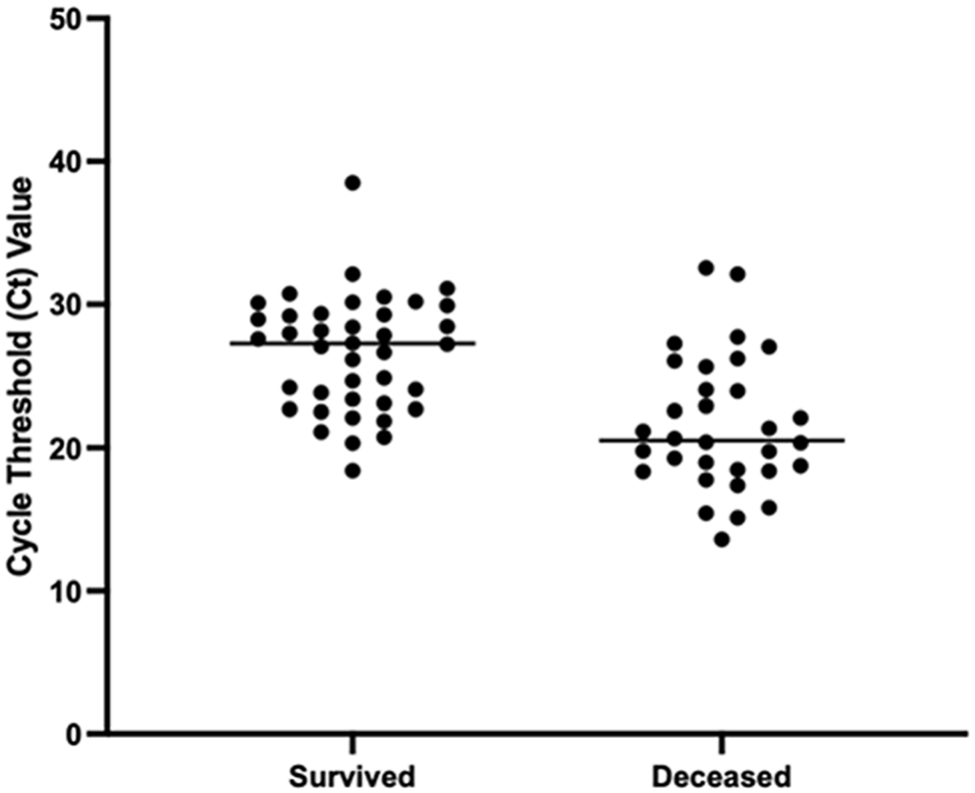
Association between Cycle Threshold (Ct) values at initial SVD diagnosis and final clinical outcome in the 72 positive cases confirmed in the mobile laboratory

A total of 72 positive cases of SUDV were confirmed by PCR in the Mubende mobile laboratory during the outbreak period. Of these, 62% were males. The mean age of SUDV positive males was 29 years (range 0–70), higher than observed in females, at 25 years (range 3–60) (see Fig. 2). Of the 72 positive cases diagnosed in the Mubende mobile laboratory, the case fatality rate was 44.5%. Analysis of cycle threshold (Ct) value as a proxy for viral load during initial laboratory testing, revealed that the median Ct value in survivors was 27.26 (range 18.41–38.50). In contrast, Ct values in the deceased individuals were lower, with median 20.53 (range 13.61–32.56), demonstrating a significant association between viral load and clinical outcome (*P* < 0.0001) (see Fig. 3).

All patients were followed up by molecular testing until either the virus in the blood was undetectable by RT-PCR or the patient was deceased. Of the survivors tested in the Mubende mobile laboratory, 23 had undetectable levels of SUDV by the first follow up test, with a median clearance time of 6 days after initial diagnosis (Table 4). Viral clearance from blood for survivors ranged from 5 days to 23 days. For patients that did not survive, the majority (81%) died within the first 2 days of diagnosis, before any follow-up tests could be conducted. All deceased patients had died within 10 days of diagnosis and overall demonstrated lower initial Ct values, indicating higher viral loads at the time of diagnosis. There appeared to be a trend between Ct value and time to death (Table 4).

**Table 4:** Days to viral clearance or death after initial SVD diagnosis in the Mubende mobile laboratory

	Follow up visits	*n*	Ct value (median (Q1 – Q3)	Days to viral clearance or death (median Q1 – Q3)
	Initial visit 0 Follow up visit 1	40 22	27.3 (23.2–29.3) 28.2 (24.4–30.1)	6 (5–8)
	Follow up visit 2	9	22.68 (20.73–26.39)	12 (5–17)
	Follow up visit 3	4	28.75 (28.2–29.9)	17 (8–18)
**Survivors**	Follow up visit 4	4	24.1 (23.4–26.9)	16 (15–18)
	Follow up visit 5	0	0	0
	Follow up visit 6	1	22.1	23
	Initial visit 0	32	20.5 (18.4–25.2)	
**Deceased**	Follow up visit 1	4	19.8 (18.7–20.9)	3.5 (2.0–19.3)
	Follow up visit 2	2	25.8	9.5

### Analysis of Sudan virus persistence in survivors

Out of the four breast feeding mothers, 3 out of 4 cleared the virus from breast milk, taking a median of 135 days after initial diagnosis. The fourth mother mirrored the general trend of viral decline in breast milk, with observed Ct values increasing from an initial value of Ct 24.76 to Ct 39.85 at the final follow up time point. The infant of one breast feeding mother was subsequently confirmed as SUDV positive and subsequently died. Sudan virus persistence in the male semen showed a similar trend to the breast milk, with 17 out of the 22 males clearing the virus. Viral clearance from semen took a median of 123 days from initial diagnosis.

**Fig. 4:**
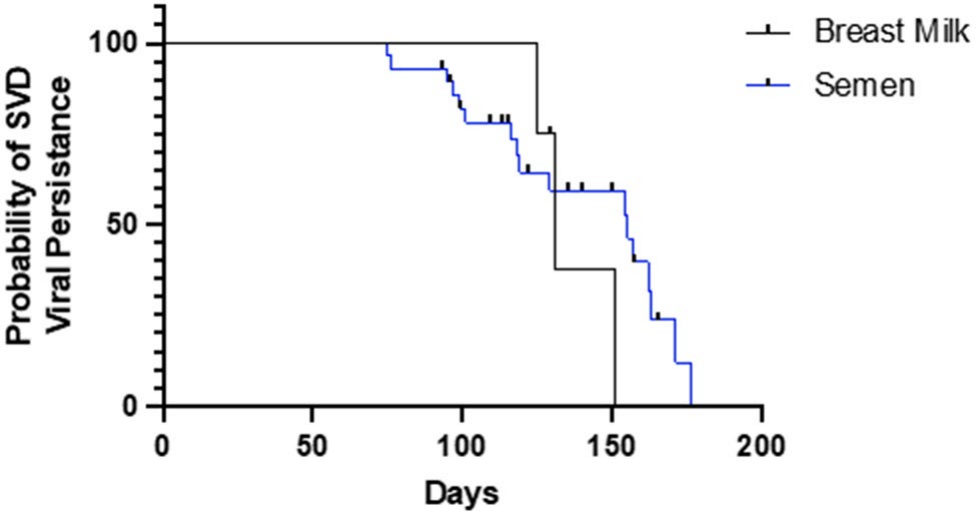
Survival analysis of Sudan virus in the semen and breast milk of survivors. Four lactating mothers and twenty-two sexually active males followed up om the mobile laboratory

Survivor analysis of viral persistence in the breast milk and semen samples, revealed that the virus appeared to be stable in breast milk for over 100 days, before rapidly declining (Fig. 4). In contrast, the virus started declining earlier in the semen, but with a more gradual decline. However, the limited female sample size relative to the male, reinforced by statistical analysis, revealed no significant difference in Sudan viral clearance between breast milk and semen groups (*P* = 0.49).

## Discussion

Between September 2022 and January 2023, Uganda experienced its sixth ebolavirus outbreak, confirmed as Sudan virus. In total, 142 cases and 55 deaths were confirmed nationally, with an additional 22 probable cases and deaths reported, giving a national CFR of 39% (excluding probable cases) [14]. Within the mobile laboratory, deployed to the outbreak epicentre of Mubende, 72 cases and 32 deaths were recorded (CFR 44%). The mobile laboratory deployment provided an opportunity to test the interoperability with the ETU and assess diagnostic turnaround time (TAT). We observed a marked reduction in diagnostic TAT, from 24 to 48 h prior to the mobile laboratory operalisation to 6 h. The close follow up of survivors in the mobile laboratory, enabled assessment of SUDV clearance from female breast milk (median 135 days) and male semen (median 123 days). Analysis of the national data revealed evidence of concurrent VHF outbreaks in Uganda, indicating a potential under-diagnosis of circulating viruses causing VHFs in the country. This underscores the importance of effective VHF surveillance in Uganda, particularly with the continuous threat of emerging zoonotic viruses [15].

### Mobile laboratory deployment

The mobile laboratory was deployed from NHLDS in just over one week of the outbreak confirmation, underscoring the readiness and integration of mobile laboratories into the national outbreak response mechanism [16]. By positioning the mobile laboratory at the epicentre of the SVD outbreak in Mubende, diagnostic TAT was significantly reduced to an average of 6 h. Previously, such highly infectious samples could only be transferred across the country from the outbreak site to UVRI, which depending on the location of the outbreak, could take in excess of 12 h to reach the laboratory and also incurred significant transportation costs. Subsequent diagnosis at the central laboratory could take a further one day, resulting in TATs of approximately 24–48 h [15]. In addition, integration of the mobile laboratory into the national outbreak response also reduced the diagnostic burden on one single facility, with the mobile laboratory in Mubende conducting 48.8% of the national testing and confirming 51% of all positive cases. Furthermore, UVRI conducted genomic sequencing on all positive samples, enabling phylogenetic analysis of the evolving outbreak to support the response, including in the mobile laboratory [17]. Having such national decentralized laboratory infrastructure in place for rapid and robust diagnosis of risk group 4 pathogens, markedly reduces the reliance of Uganda on external international laboratory support, which can take time to mobilise, can be logistically challenging and more expensive to operationalize.

### Interoperability

The mobile laboratory was set up next to the ETU in the Mubende Regional Referral Hospital and operated by a team of 18 trained personnel. This positioning enabled direct interoperability between the mobile laboratory personnel and the clinicians within the ETU. The laboratory team communicated daily with the clinical team, however weekly meetings were held to discuss sample quality, frequency of sampling for case follow- up and the interpretation of the laboratory results. The benefit of patient-centred laboratory diagnostic support has been demonstrated in previous Ebola disease outbreaks to directly impact patient outcome [18] and could explain the lower CFR observed in this outbreak (39% nationwide, 44% in Mubende mobile laboratory), compared to previous SVD outbreak estimates [6–8, 19]. This could be explained through the earlier diagnosis of patients, ebanling the clinical team to provide more timely supportive care. Furthermore, the regular reporting of viral load data to the clinical team, allowed doctors to more closely monitor disease progression and therefore be more responsive to adjust the supportive care as necessary. The higher CFR in the Mubende ETU can be attributed to the fact that it was receiving the majority of patient cases from the outbreak and that in general, the sicker patients were sent to Mubende for treatment. Nevertheless, such interoperability with clinicians is not possible if samples are tested at centralized facilities, highlighting the value of having mobile laboratory infrastructure at the site of the outbreak.

### Characteristics of SUDV positive cases

We evaluated the characteristics of the SVD cases in the Mubende mobile laboratory and nationally. The demographic characteristics were similar across the laboratories. However, we did observe an under representation of cases from the health sector in the Mubende mobile laboratory. Furthermore, with the outcome data available for the Mubende mobile laboratory, we observed that females had a poorer outcome relative to males, despite the fact that both in the Mubende mobile laboratory and nationally, there were more male positive cases. This may be for several reasons, including poorer access to health care and social behaviour in relation to burial practices (which was observed in the Kasanda district to have a direct impact on infection).

### Viral load and clearance

The ability to conduct RT-PCR in the mobile laboratory, combined with the close proximity of the mobile laboratory to the ETU, enabled regular diagnostic follow up of patients and therefore analysis of viral clearance and viral load association with patient outcome. In line with previous findings, we observed a significant association between initial Ct value (as a proxy for viral load) and patient outcome, with lower Ct values associated with worse outcome [7]. Furthermore, viral clearance from infected patient blood samples is consistent with previous studies, with the virus rarely detected by RT-PCR later than 16 days after onset of illness [20].

Understanding persistence of Sudan virus in the body fluids of convalescent patients is important, particularly in breast milk and semen, where risk of horizontal transfer of infection is possible, as previously documented in a limited number of studies [21–23]. In this current Ebola outbreak, four of the women confirmed as SUDV positive were lactating mothers at the time of diagnosis. Of these, one infant was confirmed with SUDV infection and subsequently died (although the transmission route of infant infection was not determined). In accordance with World Health Oganization (WHO) guidelines, all infected mothers were advised to stop breast feeding, to reduce the risk of onward transmission to the infants [24]. The mobile laboratory in Mubende was in the unique position to obtain breast milk samples from the four mothers and follow them up at regular time points (until 21st Mar 2023), to measure viral persistence in breast milk after demonstrated viral clearance from the serum. In all four mothers, viral RNA was still detected in breast milk samples over 100 days after clearance from the serum, supporting evidence that Sudan viral RNA can be cleared from the blood, yet persist in mammary breast tissue [20]. This is in line with previous studies, which show Ebola viral RNA can remain present in breast milk in up to 500 days after disease recovery [25, 26]. One limitation of the analysis in the mobile laboratory was that we could not determine the infectiousness of the viral RNA detected in the breast milk samples. Previous studies suggest that only around 25% of RT-PCR filovirus positive breast milk samples are infectious, indicating that positive detection of viral RNA in the breast milk alone is not enough to determine infant disease transmission risk [27, 28]. Genome sequence analysis of samples from the infected mother and infant in this current study, may elucidate further information on transmission routes, however, conclusive data on breast milk infectiousness remains limited.

Similar to the breast milk analysis, viral RNA persisted in the semen samples was in excess of 100 days post infection. This is in line with findings from both the West Africa Ebola outbreak (which due to the unprecedented scale with over 16000 survivors) provided opportunity for longitudinal semen-testing programs, and also survivor follow-up data from the DRC. This data revealed Ebola virus detection in a small number of semen samples up to 18 months post infection [29, 30]. Sequence analysis from previous studies has evidenced sexual transmission of Ebola virus in rare cases [29], highlighting the importance of the Ugandan Ministry in this current outbreak to maintain behavioral counseling for survivors, including use of barrier methods, to minimize the emergence of new transmission chains. In this current study, follow up testing within the mobile laboratory had to stop, due to re-deployment of the laboratory to the Mutukula border between Uganda and Tanzania, in response to the declared Marburg virus outbreak in the Kagera region, Tanzania, reported on 21st March 2023 [31]. At the time of mobile laboratory decommissioning, 12 out of the 29 males under follow up still had Sudan virus detected in their semen and 1 out of 4 females had virus in their breast milk. All convalescent males and females still under semen and breast milk surveillance were therefore transferred to UVRI, to continue follow-up under the Uganda Ebola disease survivor program.

### Study strengths and limitations

This study provides a detailed operational and clinical overview of the 2022–2023 Sudan virus outbreak in Uganda, uniquely integrating diagnostic, epidemiologic, and outcome data. A key strength is the use of the mobile laboratory in such an outbreak, to enable on-site molecular confirmation, interoperability and result reporting from the outbreak epicentre. This infrastructure substantially reduced diagnostic turnaround time, improved sample traceability, and supported coordinated public health decision-making. The dataset provides valuable insights into the demographic distribution, case fatality patterns, and operational efficiency of mobile laboratory deployments during this outbreak.

However, several limitations should be acknowledged. First, the study was conducted under emergency outbreak conditions, where data completeness and consistency were affected by rapid response demands. Some clinical and epidemiologic metadata were missing or incomplete, which constrained some subgroup analyses. Second, although diagnostic accuracy was high, heterogeneity in sample condition and type could have influenced molecular results. Third, the study population was geographically concentrated in the Mubende and Kasanda districts, potentially limiting the representativeness of findings across Uganda. Finally, as an observational analysis of outbreak data, causal inferences regarding determinants of disease severity or transmission cannot be made.

Despite these limitations, this investigation demonstrates the feasibility and impact of embedding high-throughput diagnostic capacity within decentralized outbreak response systems. The findings underscore the strategic value of mobile laboratory networks in strengthening epidemic preparedness and response, and they provide a framework for future One Health studies exploring host, pathogen, and environmental determinants of viral hemorrhagic fevers in East Africa.

## Conclusion

The Sudan virus outbreak was the second largest Ebola outbreak on record in Uganda, with 142 confirmed cases and 55 confirmed deaths. Given the spread of cases to the capital, Kampala, the potential for an epidemic (similar that experienced in West Africa), was a possibility. The rapid deployment of the mobile laboratory to the outbreak epicentre in Mubende, as part of the national response, ensured faster molecular diagnosis of cases to support rapid containment measures. It also removed the reliance on external international mobile laboratory support, which can take time to mobilise. Furthermore, removing long sample shipments required for centralized testing, reduced the associated biosafety risks and potential compromises to sample integrity, which can impact diagnostic results. The mobile laboratory demonstrated the benefit of interoperability with ETUs for rapid containment and continuous patient care and furthermore, the results from the mobile laboratory have contributed to a greater understanding of SUDV. In addition, the redeployment of the mobile laboratory in response to the Tanzanian Marburg outbreak, highlights the versatility of the mobile laboratory to operate in both response mode at the outbreak epicentre and in preparedness mode. The Uganda Ministry of Health has therefore demonstrated the value of integrating mobile laboratories into National public health emergencies, which is a model that can be replicated by other Ministries of Health.

Since 30th January 2025, Uganda is once more facing an Sudan virus outbreak in the country [32]. Identified in the Capital Kampala, this is the seventh Ebola disease outbreak in recent years. Leveraging on the capacity built up during the previous SVD outbreak, mobile laboratories are once again active in this national response.

## Data Availability

The personal details datasets used for this descriptive analysis are a property of the Uganda MoH and are not publicly available. However, with a reasonable request and permission from Uganda MoH, data can be accessed through the corresponding authors.

## Abbreviations

SUDV: Sudan Virus
ED: Ebola Disease
MoH: Ministry of Health
NHLDS: National Health Laboratory and Diagnostics Services
VHF: Viral Haemorrhagic Fever
ETU: Ebola Treatment Unit
TAT: Turn-Around-Time
EVD: Ebola Virus Disease
EBOV: Ebolavirus
TAFV: Tai Forest virus
BDBV: Bundibugyo virus
DRC: Democratic Republic of the Congo
CFR: Case Fatality Rate
RRH: Regional Referral Hospital
UVRI: Uganda Virus Research Institute
RT-PCR: Real Time Polymerase Chain Reaction
RVF: Rift Valley Fever
CCHF: Crimean Congo Haemorrhagic Fever
CPHL: Central Public Health Laboratory
RDS: Results Dispatch system
Ct: Cycle Threshold
EAC: East African Community
WHO: World Health Organization
